# Code Help: Can This Unique State Regulatory Intervention Improve Emergency Department Crowding?

**DOI:** 10.5811/westjem.2018.1.36641

**Published:** 2018-03-08

**Authors:** Sean S. Michael, John P. Broach, Kevin A. Kotkowski, D. Eric Brush, Gregory A. Volturo, Martin A. Reznek

**Affiliations:** * University of Massachusetts Medical School, Department of Emergency Medicine, Worcester, Massachusetts; † University of Colorado School of Medicine, Department of Emergency Medicine, Aurora, Colorado

## Abstract

**Introduction:**

Emergency department (ED) crowding adversely affects multiple facets of high-quality care. The Commonwealth of Massachusetts mandates specific, hospital action plans to reduce ED boarding via a mechanism termed “Code Help.” Because implementation appears inconsistent even when hospital conditions should have triggered its activation, we hypothesized that compliance with the Code Help policy would be associated with reduction in ED boarding time and total ED length of stay (LOS) for admitted patients, compared to patients seen when the Code Help policy was not followed.

**Methods:**

This was a retrospective analysis of data collected from electronic, patient-care, timestamp events and from a prospective Code Help registry for consecutive adult patients admitted from the ED at a single academic center during a 15-month period. For each patient, we determined whether the concurrent hospital status complied with the Code Help policy or violated it at the time of admission decision. We then compared ED boarding time and overall ED LOS for patients cared for during periods of Code Help policy compliance and during periods of Code Help policy violation, both with reference to patients cared for during normal operations.

**Results:**

Of 89,587 adult patients who presented to the ED during the study period, 24,017 (26.8%) were admitted to an acute care or critical care bed. Boarding time ranged from zero to 67 hours 30 minutes (median 4 hours 31 minutes). Total ED LOS for admitted patients ranged from 11 minutes to 85 hours 25 minutes (median nine hours). Patients admitted during periods of Code Help policy violation experienced significantly longer boarding times (median 20 minutes longer) and total ED LOS (median 46 minutes longer), compared to patients admitted under normal operations. However, patients admitted during Code Help policy compliance did not experience a significant increase in either metric, compared to normal operations.

**Conclusion:**

In this single-center experience, implementation of the Massachusetts Code Help regulation was associated with reduced ED boarding time and ED LOS when the policy was consistently followed, but there were adverse effects on both metrics during violations of the policy.

## INTRODUCTION

Emergency department (ED) crowding adversely affects at least two of the Institute of Medicine’s (IOM) domains of high-quality care: safety and timeliness.^1^,^2^ While the causes of ED crowding are multi-factorial, “output” (flow of admitted patients into inpatient settings) is cited as a leading cause.^3^,^4^ The IOM identifies the phenomenon of holding patients in the ED after the decision to admit (known as boarding) as a public health crisis and has urged hospitals and accrediting bodies to improve inpatient resources and flow to reduce boarding of patients in the ED.^4^ Advocates recommend a number of countermeasures to improve the flow of boarded patients, and a common theme among them is the importance of recognizing that the flow of ED patients is a systemic, hospital-wide issue, rather than a problem localized to the ED.^4^–^6^ Sporadic adoption of recommended ED-boarding countermeasures to date has led some authors to suggest that “enhanced regulation” may be required if current strategies fail to reduce boarding.^7^,^8^

It appears the Commonwealth of Massachusetts’s Department of Public Health (DPH) is the first state regulatory body to mandate specific, hospital action plans to reduce ED boarding via its “Code Help” concept.^9^ A number of regulatory and state agencies support efforts to reduce ED boarding by permitting inpatient floor boarding or mandating reporting of ED flow data, but none except for the Massachusetts DPH appear to have mandated specific, hospital action plans with pre-defined triggers.^9^,^10^

In December 2002, as part of a multi-pronged attempt to eliminate ambulance diversion, the DPH sent a letter to Massachusetts hospitals that included a mandate to develop individual hospital “Code Help” policies with “provisions to redeploy hospital staff and resources with a goal of moving all admitted patients out of the ED within 30 minutes [of activation].”^11^ When Massachusetts became the first state to ban ambulance diversion,^12^ the DPH stipulated that Code Help policies must include escalation to “appropriate emergency management/disaster plans and protocols” should the initial actions not adequately decompress the ED within two hours. Hospitals were required to submit their Code Help plans for review in early 2010, and the DPH’s subsequent assessment revealed that many of the plans were inadequate and lacked the specificity required by regulation.^11^ In 2015, the DPH re-emphasized the importance of Code Help and insinuated that plans would be reviewed as part of their routine, hospital-survey processes.^11^

Despite the DPH’s consistent emphasis on compliance with the Code Help countermeasure, boarding continues to be a critical issue across the Commonwealth,^11^ raising questions as to the effectiveness of the Code Help initiative. As a part of process improvement efforts for our institution, one author (MAR) solicited informal feedback from Massachusetts ED directors regarding the application and effectiveness of Code Help at other institutions. Most respondents reported no qualitative improvement in ED flow after creating Code Help policies at their hospitals. Some added that Code Help was inconsistently applied and suggested that this contributed to its lack of effectiveness Martin A. Reznek, (unpublished personal communication).

**Population Health Research Capsule**

What do we already know about this issue?

Emergency department (ED) overcrowding adversely affects quality and patient safety, but countermeasures are limited. Massachusetts mandated hospital action plans (“Code Help”), the impact of which is unknown.

What was the research question?

Does Code Help mitigate adverse effects of overcrowding by reducing boarding time and ED length of when the policy is followed?

What was the major finding of the study?

Code Help implementation is associated with shorter ED boarding time and length of stay when the policy is consistently followed.

How does this improve population health?

If the effects of this single-center experience are replicated more broadly, mandates on hospitals may have potential to decrease patients’ exposure to the negative effects of overcrowding.

Anecdotal experience at our institution was similar, and we found adherence to an effective Code Help procedure to be historically difficult and inconsistent. However, as the policy gained broader acceptance from hospital leadership, we saw an opportunity to evaluate whether Code Help is effective when completely implemented and the DPH guidelines followed. We hypothesized that compliance with the Code Help policy would be associated with reduction in ED boarding time and admitted-patient total ED length of stay (LOS), compared to patients seen when the Code Help policy was not followed.

## METHODS

### Study Design and Setting

This was a cohort study conducted at a 364-bed urban, academic, tertiary referral center with trauma, stroke, and cardiac programs serving approximately 27,000 adult inpatients annually, with 65,000 annual adult ED visits and a monthly ED adult admission rate of 26–30%. There is a co-located pediatric center, but it is operationally distinct and includes a separate pediatric ED.

The hospital developed and implemented its Code Help policy in accordance with the DPH mandate and guidelines. The policy includes standardized triggers, activation processes, next steps in the event of failure, and testing and evaluation as outlined by the DPH.^11^ In general, three levels of activation were observed: “normal operations;” “Code Help;” and escalation to the hospital emergency operations plan (also known as “disaster plan activation”). The hospital disaster plan required in-person or conference-call response of all hospital managers, use of the Hospital Incident Command System, including a defined incident commander, conducting regularly scheduled briefings, and a continuously operational Emergency Operations Center where resources and decision-makers for the hospital system are located. Table 1 includes relevant text from the Code Help policy.

### Measurements and Selection of Participants

We created a prospective Code Help event registry on October 1, 2014, enabling ascertainment of Code Help event timestamps following that date. We retrospectively queried the electronic health record (EHR) for consecutive individual patient visits of all adult ED patients from October 2014 through January 2016. For all admitted patients, we extracted EHR timestamps tracking four patient flow events: ED arrival, ED triage completion, admission decision, and ED departure time (physically moved to an inpatient unit). The electronic inpatient bed request placed by the ED provider following a verbal acceptance of the patient by the admitting team served as a proxy for admission decision.^13^ We included patients admitted to either a medical/surgical acute care hospital bed (including telemetry) or critical care bed. Patients admitted to psychiatry, labor and delivery, or directly to a procedural area (operating room or cardiac catheterization lab) were excluded, as the EHR admission timestamp data were known to be unreliable for these patients due to unique admission processes related to those units. We defined boarding patients as those who remained in the ED after the decision to admit and defined boarding time as the interval between the admission-decision time and the departure time from the ED.^10^ We defined total ED LOS as the interval between ED arrival time and physical departure time from the ED.^10^

### Code Help Exposure Status

We matched each patient visit against our prospectively collected registry of Code Help events, which contained start and stop times for each Code Help event as well as hospital disaster-plan activation time, if applicable. For each patient, we determined the hospital’s concurrent Code Help “status” (normal operations, Code Help, or disaster plan) at the time of each of four patient flow events: ED arrival time, ED triage time, admission decision time, and ED departure time.

We then determined whether the concurrent hospital status complied with the Code Help policy or violated the policy at the time of each patient flow event. Any of three possible scenarios constituted a policy violation: (1) the ED operational environment met Code Help activation criteria, but Code Help was not activated; (2) Code Help criteria had been met for greater than two hours without escalation to the hospital disaster plan; or (3) Code Help was re-activated within 24 hours without escalating directly to the hospital disaster plan. We determined the latter two violations by using the Code Help registry timestamps to calculate the elapsed time in the Code Help status and the elapsed time since the last Code Help/disaster event, respectively. Determination of the first violation type (the ED operational environment met criteria for Code help activation but remained in normal operations) required a standardized measure of the state of operations and flow in the ED.

Recognizing inherent limitations of all current quantitative measures of ED crowding, we selected ED occupancy ratio (EDOR), the number of patients currently in the ED divided by the number of licensed ED treatment spaces, as a surrogate for ED resource demand due to its prior use in the literature and relative ease of calculation as an instantaneous measure.^14^–^22^ An EDOR greater than 100%, by definition, would fulfill the Code Help activation criterion of “capacity of the ED exceeds licensed bed capacity” (Table 1).^11^ However, our ED routinely operated with staffed, unlicensed “hallway” beds, and the number of these beds varied in response to patient demands and resource availability. As such, an EDOR of 100% would accurately reflect the ED licensed bed capacity but would underestimate our functional ED capacity.

We had no way to determine the exact number of unlicensed, staffed treatment spaces at any given time, so we sought to identify a surrogate EDOR threshold to more accurately reflect our functional ED capacity limit. Our initial analysis suggested that EDOR of 200% corresponded to the 99th percentile for all hours during the study period. Further, we verified that for each hour where EDOR exceeded 200%, there was at least one boarded patient in the ED (minimum 5, median 21, interquartile range [IQR] 9), which fulfilled the second trigger criterion for Code Help (Table 1). We categorized any time during which EDOR exceeded 200%, but neither Code Help nor the disaster plan were active, as being a probable violation of the policy. We validated this approach against an alternative logistic regression model (see Appendix).

### Statistical analysis

To assess the effects of compliance and non-compliance with the Code Help policy, we performed univariate comparisons of boarding time and overall ED LOS for patients cared for during periods of Code Help policy compliance and during periods of Code Help policy violation, both with reference to patients cared for during normal operations, using Steel’s method, the nonparametric version of Dunnett’s test, which controls the error rate for multiple comparisons vs. the control group.^23^ We chose to compare each scenario to a common reference standard (normal operations) because doing so improved the overall error rate compared to pairwise comparisons and allowed us to evaluate the efficacy of Code Help in maintaining patient flow as close to normal operations as possible, despite the crowding and adverse circumstances that triggered Code Help activation. Performing only a direct comparison between policy compliance and policy violation would have ignored the valuable data from the large number of patients seen during normal operations, who could serve as a common control group, and would have dramatically reduced our statistical power to identify a between-group difference because of the reduction in population size.

Using the same technique, we performed a secondary analysis of the same metrics during any Code Help event or disaster activation (regardless of policy compliance), with reference to patients cared for during normal operations. We felt this secondary analysis was important to evaluate the effects of Code Help/disaster itself, even if misapplied or inconsistently followed. We also performed a number of sensitivity analyses to validate our analytic choices (see Appendix). Analyses were conducted using JMP Pro 12 (SAS Institute Inc., Cary, NC). The study was approved by our institutional review board.

## RESULTS

### Characteristics of Admissions and Code Help Events

Of 89,587 adult patients who presented to the ED during the study period 26,065 (29.1%) were admitted, 24,017 (92.1%) of whom were admitted to either an acute care or critical care bed and included in further analysis. Of the admitted patients, the median age was 64 (IQR 26), and 48% were female. Boarding time ranged from zero to 67 hours 30 minutes (median 4 hours 31 minutes) and was less than two hours for 14.2% of admitted patients. Total ED LOS for admitted patients ranged from 11 minutes to 85 hours 25 minutes (median 9 hours). ED occupancy ratio at the time of decision to admit ranged from 34% to 243% (mean 128%, standard deviation 33) and was stable over the time period of the study.

There were 89 Code Help events recorded in the registry during the study period (every 5.4 days on average), and 23 (26%) progressed to disaster plan activation. The probability of progressing to disaster plan increased over time, while the monthly frequency of Code Help events decreased (Figure). Time from Code Help activation until disaster activation ranged from 57 minutes to 3 hours 25 minutes (median 2 hours 39 minutes), and there were 64 instances of not escalating to the hospital disaster plan, despite meeting the two-hour criteria.

### Policy Violations

Among all admitted patients, 2,219 (9.2%) had a decision to admit during Code Help, and 492 (2.0%) had a decision to admit during disaster. Among these 2,711 patients, 1,383 (51%) were admitted during a policy violation (1,227 while Code Help had been active greater than two hours, but the disaster plan had not yet been activated, and 156 during a re-activation of Code Help within 24 hours without activating the disaster plan). We identified an additional 94 patients admitted during a presumptive policy violation, where EDOR exceeded 200% but neither Code Help nor the hospital disaster plan were active.

### Code Help Effectiveness

Each Code Help event was associated with a mean 17% reduction (95% CI [12%–22%]) in the number of patients boarding at the end of Code Help, compared to the time of activation. However, much of this reduction was accomplished after the first 30 minutes of Code Help, despite the stated policy goal of removing all boarding patients from the ED within 30 minutes. In the first 30 minutes, there was a mean 0% reduction in boarding patients (95% CI [3.4% increase to 0.2% decrease]).

### Main Results

When not accounting for policy compliance, median boarding time and total ED LOS were longest during disaster activation and shortest during normal operations (Table 2). However, when accounting for Code Help policy compliance vs. violations of the policy, patients admitted during periods of any type of Code Help policy violation had significantly longer boarding times and total ED LOS, compared to patients admitted under normal operations (Hodges-Lehmann estimate of 25 minutes [95% CI {13–37 minutes} of additional boarding time and 45 minutes [95% CI {26 minutes to 1 hour 5 minutes}] of additional ED LOS). Among patients admitted during periods of Code Help policy compliance, in contrast, we found no significant difference in either metric, compared to normal operations. Table 3 reports the distributions of each metric for each subgroup.

### Sensitivity Analyses

Our results were insensitive to the choice of patient flow-event timestamp linkages. Of the four events, we selected decision to admit for the primary analysis because we presumed that Code Help countermeasures were likely to have the greatest potential impact on a patient’s flow into the inpatient setting if active at the time of the decision to admit. Lagged effects of Code Help were maintained at 30 and 60 minutes after the end of a Code Help or disaster event, but effects did not persist at 90 minutes or six hours. Our results were substantially unchanged when considering only the second two policy-violation types, discarding the EDOR threshold.

## DISCUSSION

Our results suggest that when the Code Help concept is implemented in a manner that complies with DPH requirements and the policy is followed, both ED boarding time and total ED LOS for admitted patients appear to be reduced to durations typical of normal operations, despite increased ED demand. Violations of the Code Help policy appear associated with the loss of those benefits. We observed a 14-minute relative increase in median boarding time and a 28-minute increase in ED LOS among patients admitted during periods of policy violation, compared to those admitted during periods of policy-compliant Code Help or disaster, despite equally adverse ED operational conditions. This difference cumulatively represents approximately 689 patient-hours (28.7 patient-days) of ED capacity during the study period, which would otherwise have been available to care for additional ED patients had the policy been followed.

Our results suggest that compliance with the Code Help policy is pivotal in achieving improved ED flow, rather than simply having the policy in place but not following its guidelines. The DPH’s Code Help concept, when implemented correctly and consistently, may reduce ED boarding and crowding and represents an important countermeasure to supplement the relatively limited armamentarium of current strategies.^5^,^7^ It is worth noting, however, that even when the policy is followed Code Help does not appear to achieve its stated objective of removing all boarding patients from the ED within 30 minutes of activation.

While the overall results of this study are encouraging, it is not clear what specific factors within the Code Help policy implemented at our institution led to flow improvements. We believe that its effectiveness lies in the fact that the policy sets clear expectations, has a defined escalation process, requires hospital-wide leadership involvement, and establishes real-time accountability. It mandates action by leaders outside of the ED, who can problem-solve on a system level and engage in real-time, team-based solutions, and it provides a standardized structure for how to do so. At its core, Code Help provides for a hospital-wide response to a hospital-wide patient flow problem, even if the primary manifestation of that problem appears only in the ED.

One shortcoming of the Code help concept is that it focuses on reactive, rather than proactive, responses to crowding. While lessons learned during each Code Help activation may result in incremental process improvements during normal operations, Code Help actions do little to directly smooth flow or increase throughput when the plan is *not* active. Another disadvantage of the Code Help concept is the potential frequency with which the hospital disaster plan must be activated in the event Code Help is ineffective after two hours. When Code Help is routinely activated, the demand for frequent briefings and conference calls may compete with hospital leaders’ other duties. This may raise awareness regarding crowding in the short term, but other long-term priorities may be inadvertently adversely affected.

To our knowledge, the DPH Code Help regulation is the first of its kind in the U.S. that mandates specific hospital actions to alleviate ED boarding.^9^ The Centers for Medicare and Medicaid Services now requires reporting of ED flow measures, and the Joint Commission requires hospitals to have committees that oversee hospital flow, but neither mandates specific, ED-boarding countermeasures.^24^ The DPH Code Help initiative presents a unique opportunity to evaluate whether “enhanced regulation” may reduce ED boarding, as suggested in prior literature.^7^ While it remains to be seen if the DPH Code Help regulation will be successful across the Commonwealth over time, the results of this study suggest that it may be effective if hospital policy meets the DPH requirements and is followed consistently.

## LIMITATIONS

Given the potential confounders and time-dependent nature of this dataset, we considered a number of analytic approaches and found that each approach, including our final analysis plan, had substantial limitations. ED LOS and boarding time are time-to-event data. Although they do not exhibit censoring (i.e., we have available LOS data for each patient, no matter how long they waited for admission), our observed boarding times do have some similarities to survival data, in that the probability of a given patient remaining in the ED at any point in time is conditional on the patient’s presence in the ED during all preceding times since their arrival. Thus, we considered using Cox proportional hazards regression, but our dataset did not seem to fit the assumption that the “hazards” (that is, the probability of a given patient ending their ED LOS in the next minute) are strictly proportional between groups. We also considered a time-series approach, which still did not completely address our limitations. Our simpler approach of comparing group medians and distributions was less powerful, but we felt more assured that our data satisfied the prerequisites of the more conservative Steel test.

Comparisons of group medians, however, are inherently disadvantageous, in part because our method of stratifying patients to policy-compliant and policy-violation groups insinuates that there is a clear delineation between these cohorts. In fact, there is very little discernable difference between a patient admitted 119 minutes into a Code Help event (technically policy-complaint) and a patient admitted at 121 minutes without disaster plan escalation (a policy violation). Similarly, while the operational environment when the EDOR is 195% is quite similar to that when the EDOR is 205% when Code Help is not active, our approach would trigger a policy violation only for the latter. It is implausible that Code Help would have a differential effect at EDOR 205%, compared to 195%, or 119 minutes, compared to 121 minutes, but our analytical approach assumes that it may.

A related limitation is that detecting failure to activate Code Help when criteria were initially met required a surrogate marker because of the retrospective nature of the investigation. Because ED census exceeding licensed ED bed capacity was a criterion for Code Help activation, EDOR was a natural choice as a marker. Our data demonstrated that there were always admitted patients boarding in the ED when EDOR exceed 102%. However, it was also the case that our usual operations included evaluating and treating patients in staffed but unlicensed hallway spaces, so 100% occupancy is likely an overestimate of functional crowding in our ED. Our threshold of 200% (99^th^ percentile of EDOR) was intended to be conservative and more specific than sensitive. By design, the risk of falsely categorizing a patient as having been admitted during a policy violation was low, but we likely failed to identify some true violations that may have occurred at times when EDOR was between 102% and 200%. This type of violation accounted for only 11% of the patients admitted during any policy violation and probably underestimates actual violations. In our post-hoc sensitivity analysis to consider a comparison of only absolute policy violations (by considering the 94 patients admitted with EDOR >200% to be in the normal operations group), our findings were substantially unchanged.

We also had no mechanism to measure overall hospital demand-capacity mismatch outside of the ED nor insight into the specific decision-making that resulted in Code Help policy violations. Consequently, it is possible that violations were associated with hidden, external factors, such as leaders sensing complete hospital resource saturation and not following the policy due to feelings of futility. Further, a prospective power analysis was not possible given the study design. It is conceivable that any of these factors may have resulted in our failure to detect a difference in outcomes between the policy-compliant and normal operations groups, where one existed in reality (a Type II error). Nevertheless, the magnitude of difference between the policy-compliant and policy-violation groups and the fact that metrics were worse under Code Help when ignoring policy compliance (Table 2) suggest that policy compliance likely has a real differential effect.

Finally, our study reports the experience of a single center, which naturally limits the generalizability of our findings. It is likely that the specific interventions that occur during Code Help/disaster at our institution may not be as effective at other sites because they were designed to fit our local work environment and processes. However, a key strength of the DPH Code Help concept may be that, while it does call for adherence to specific guiding principles, it does not mandate specific tactics. We believe the general principles set forth in the regulations are generalizable to all hospitals, even if they require different implementation tactics. In fact, the failure to customize these specifics to each institution’s unique workflow may be partially responsible for the initially slow adoption of the Code Help concept more generally. Based on the DPH’s own assessment, Code Help has not been effective Commonwealth-wide,^11^ but its analysis suggests this may be due to the fact that many individual hospital policies do not meet DPH requirements. Currently, we are unaware of any penalties levied by the DPH against hospitals that do not comply with the Code Help requirements. Our study may lend credence to the idea that regulators should value actual policy compliance, as opposed to hospitals simply having created a Code Help policy.

## CONCLUSION

In our single-center experience, implementation of the DPH Code Help regulation is associated with shorter ED boarding time and ED length of stay when the policy is consistently followed. However, our analytic approach has important limitations that necessitate cautious interpretation of our findings. It remains to be determined whether the regulation will result in improved outcomes more broadly across Massachusetts.

## Supplementary Information



## Figures and Tables

**Figure:**
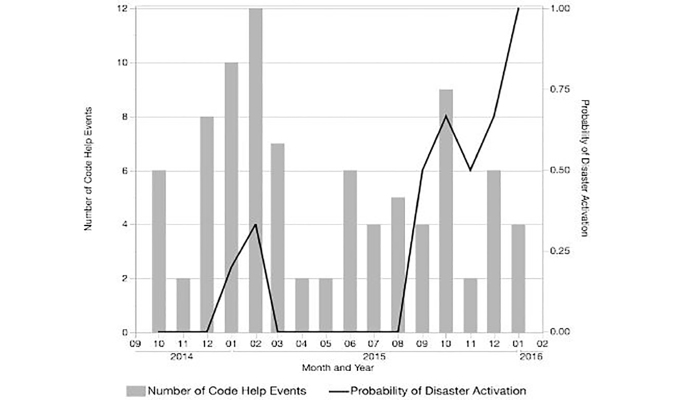
Code Help trends over time.

**Table 1:** Text of the Code Help policy.

Triggers for Code Help Code Help will be activated when the capacity of the ED exceeds licensed bed capacity, there are admitted patients boarding in the ED, and there are no licensed spaces available to see the next patient. Procedure for activating Code Help The ED Flow/Resource RN [Charge Nurse] or ED attending physician will consult ED Nursing Leadership and/or ED Administrator on-call (AOC). ED Nursing Leadership will contact the ED AOC (or vice versa) to review the current status of the ED and to determine if any other actions can be taken prior to activation of Code Help to immediately decompress the Emergency Department. Should it be determined by the above group that the ED meets Code Help trigger criteria, the ED AOC will activate Code Help by contacting the Care Connection Center [hospital transfer center]. The Care Connection Center will: Activate Code Help by sending the scripted message to all on the global address listing “Code Help” distribution list. This message will run at initial activation only. Upon receiving Code Help notification, all departments will react according to their standard work for Code Help. The Code Help Leadership Team [ED nursing and physician leaders, transfer center staff, bed assignment staff, hospital nursing supervisor] will meet within 30 minutes of activation to review the response effectiveness, additional resources needed, and next steps. Reassessment, escalation and termination of Code Help ED status will be reassessed every hour from Code Help activation by the Code Help Leadership Team. A decision will be made to continue, escalate, or stand down from Code Help status. When the burden of admitted patients has eased, the Code Help Leadership Team will come to an agreement on ‘Standing Down’ from Code Help status. If all agree, they will contact the Care Connection Center to announce “Stand Down” of Code Help. The Care Connection Center will send ‘Standing Down’ email/text page to the Code Help distribution list. Should ED Capacity exceed licensed beds within 24 hours of Code Help activation, reactivation of Code Help is not considered an adequate response. Escalation of Code Help If Code Help does not eliminate the burden of admitted patients in the ED within two (2) hours of activation, [if Code Help has been activated in the prior 24 hours,] or if the severity of the initial situation warrants it, the Code Help Leadership Team will contact the hospital AOC, COO, CNO, CMO, and President and notify them of ED status and the need to activate the hospital Emergency Operations Plan. The Hospital President or Administrator On Call will activate the Hospital Emergency Operations Plan Phase I using the following steps: Notify the Hospital Telecommunication Console operator Declare “Phase 1 of the Emergency Operations Plan is now in effect” The telecomm console will initiate activation of the overhead disaster announcement. They will then conference the caller with Public Safety Console to activate communicator message for “Phase 1 of Emergency Operations Plan activation” Command Centers will be opened and Incident Command will be established. The Command Center will refer to Annex M for roles and responsibilities related to Capacity Emergency Response Plan. Standing down Phase 1 of the Emergency Operations Plan is determined by the incident commander in consultation with the ED AOC, ED Nursing Leadership, ED physician, and Nursing House Supervisor who will review the status of the ED. If the ED is no longer within Code Help criteria the organization will stand down from the Capacity Emergency Response Plan. The notification for “Standing Down” will be made via the same process as the activation. Testing and after action review The Code Help policy will be tested during the months of January and July, unless it has been activated within the previous 6 months. An after-action review will be completed and documented for each activation and test. Written notes to be retained by Flow Leadership Committee.

Source: UMass Memorial Medical Center Policy 2246. Reprinted with permission.

**Table 2:** Boarding time and total emergency department (ED) length of stay by department status at the time of admission decision.

	Minimum	25th Percentile	Median	75th Percentile	Maximum
	Boarding time (hours:minutes)				
Normal operations (n=21,306)	0:00	2:40	4:31	8:05	67:30
Code Help (n=2,219)	0:02	2:55	4:39	8:41	43:58
Disaster (n=492)	0:23	2:54	4:51	9:14	46:45
	Total ED length of stay (hours:minutes)				
Normal operations (n=21,306)	0:11	6:08	8:57	13:53	85:25
Code Help (n=2,219)	0:49	6:33	9:23	14:30	59:27
Disaster (n=492)	0:55	6:42	9:30	15:39	67:58

**Table 3:** Boarding time and total emergency department (ED) length of stay by Code Help policy compliance at the time of admission decision.

	Minimum	25th Percentile	Median	75th Percentile	Maximum
	Boarding time (hours:minutes)				
Normal operations (n=21,692)	0:00	2:40	4:30	8:04	67:30
Policy-complaint (n=826)	0:02	2:55	4:36^NS^	7:56	46:45
Any policy violation (n=1,477)	0:03	2:56	4:50^a^	9:15	43:09
	Total ED length of stay (hours:minutes)				
Normal operations (n=21,692)	0:11	6:08	8:56	13:52	85:25
Policy-complaint (n=826)	0:49	6:33	9:14^NS^	13:49	67:58
Any policy violation (n=1,477)	0:57	6:39	9:42^a^	15:05	56:46

a p<0.001 for difference from normal operations,

NS p>0.05 for difference from normal operations.
