# Supplementary material for: Code Help: Can This Unique State Regulatory Intervention Improve Emergency Department Crowding?
Source: West J Emerg Med. 2018 Mar 8;19(3):501–9. doi: 10.5811/westjem.2018.1.36641 (PMC5942017; doi:10.5811/westjem.2018.1.36641)
Supplement: Supplementary file 1 [file wjem-19-501-s001.docx]

**Code Help: Can this unique state regulatory intervention improve emergency department overcrowding?**

**APPENDIX**

**Additional Methods (Sensitivity Analyses)**

To further validate our choice of the ED occupancy ratio (EDOR) threshold during preliminary analysis, we also constructed a logistic regression model of the probability of Code Help or disaster plan activation using EDOR (as a continuous predictor), hour of day, day of week, number of patient arrivals in the prior hour, and interaction and response surface terms as predictors. There was not a significant difference in misclassification rate between the regression model and the much simpler EDOR threshold of 200%, so we selected the latter for use in further analyses.

We also performed sensitivity analyses to determine the effect of the choice of patient flow event linkage (arrival, triage, admission decision, or departure time) on our findings, as well as potential lagged effects of a Code Help or disaster activation in 30 minute intervals from 0 to 6 hours after each Code Help or disaster event. Given the complexity of identifying potential policy violations based only on an EDOR threshold, we also performed sensitivity testing in which we repeated the primary analysis considering only the second two violation types (Code Help criteria had been met for greater than two hours without escalation to the hospital disaster plan or Code Help was re-activated within 24 hours without escalating directly to the hospital disaster plan) and considering patients admitted with EDOR >200% to be normal operations, not a probable violation.
